# Using Telehealth to Provide Interventions for Children with ASD: a Systematic Review

**DOI:** 10.1007/s40489-021-00278-3

**Published:** 2021-07-16

**Authors:** Yanicka L. de Nocker, Christina K. Toolan

**Affiliations:** Center for Autism Research and Treatment, University of California Los Angeles (UCLA), 300 UCLA Medical Plaza, Box 956967, Los Angeles, CA 90095 USA

**Keywords:** Systematic review, Telehealth, Autism spectrum disorder, Online training

## Abstract

As the need for accessible interventions for autism spectrum disorder (ASD) grows, empirically supported telehealth interventions become increasingly necessary. With the current COVID-19 public health crisis, in-person interventions have become largely infeasible; therefore, it is crucial that providers have information regarding the effectiveness of telehealth interventions. This systematic review evaluates and synthesizes existing group design research on telehealth ASD interventions. Sixteen articles were evaluated on implementer and child-level intervention outcomes as well as factors that promote equitable access to intervention. Findings suggest that telehealth programs are highly acceptable, comparable to face-to-face interventions, and can be an effective method of training implementers in interventions. Recommendations for future research and for maximizing equitable access to telehealth interventions are presented.

The prevalence of autism spectrum disorder (ASD) has risen over the last several decades, now affecting 1 in 59 children in the USA (Baio et al., 2018). As this prevalence has increased, so has the need for services to assist individuals with ASD. However, empirically supported interventions can be difficult to access, due to long waiting lists and the lack of intervention specialists, particularly in rural or underserved communities (Belfer & Saxena, 2006). Many families in these areas must manage expensive travel or equipment costs to obtain timely intervention (Wacker et al., 2013a).

ASD services have become exceptionally difficult to access at the current time, due to guidelines put in place worldwide to slow the spread of COVID-19. The Centers for Disease Control and Prevention (CDC) have recommended the reduction of group gatherings, dismissal of in-person school and extracurricular activities, implementation of telework practices, and cancellation of non-essential travel (CDC, 2020). State governments have ordered at least 316 million people in 42 states to stay home (Mervosh et al., 2020). Applied behavior analysis (ABA) as an ASD intervention (Baer et al., 1968) has been classified as an essential health service in many states (Snider, 2020), and therefore many ABA service providers have been permitted to operate as usual. Still, amid growing concerns from families and behavioral therapists, as well as increasingly strict governmental restrictions, the field of ASD intervention has become eager for a more accessible option.

One strategy that has been used to combat the issue of lack of accessibility across healthcare fields is the use of telehealth practices. Telehealth (also known as “telepractice” or “telemedicine”) allows specialists and care providers to deliver interventions remotely, using communication technology such as the internet (Bearss et al., 2018). Telehealth is already common practice in several branches of medicine (e.g., Dorsey & Topol, 2016; Webb et al., 2010) and psychology (e.g., Kessler et al., 2009). During the COVID-19 pandemic, certain health clinics have observed a marked increase in telehealth services and a decrease in in-person visits (Baum et al., 2021). It is clear that telehealth as a form of health care delivery is on the rise, and its application in hospitals, private clinics, and homes is rapidly growing.

Telehealth practices for ASD intervention and assessment are also increasing, and a growing body of literature has examined both the feasibility and effectiveness of these methods of delivery (Boisvert et al., 2010; Ferguson et al., 2019; Meadan & Daczewitz, 2015; Sutherland et al., 2018; Tomlinson et al., 2018). Most telehealth programs in this field rely on behavior analysts, psychiatrists, psychologists, education specialists, and/or university-based researchers to provide training and supervision to teachers, therapists, or caregivers of children with ASD (Boisvert et al., 2010). Training and supervision provided via telehealth has included services such as conducting functional behavior assessments (e.g., Wacker et al., 2013b), applying preference assessments (e.g., Machalicek et al., 2009), and implementing behavioral interventions (e.g., Vismara et al., 2013). ASD intervention via telehealth is not only an effective method of teaching interventions and assessments to others (Ingersoll et al., 2017; Kaiser et al., 2000), but is also largely effective in improving a number of child outcomes — namely, reducing challenging behavior (e.g., Lindgren et al., 2016) and improving communication skills (e.g., Baharav & Reisier, 2010; Vismara et al., 2013).

However, one limitation of telehealth-based ASD interventions is that the evidence base for these interventions for children is largely made up of single-subject research designs (SSRDs), as noted in several previous reviews (Boisvert et al., 2010; Ferguson et al., 2019; Knutsen et al., 2016; Tomlinson et al., 2018). SSRDs are useful for testing initial intervention efficacy and can demonstrate intervention effectiveness and generalizability with careful and systematic replication; however, they are inherently limited by small sample sizes. Group designs may build on single-subject contributions, allowing researchers to understand feasibility and effectiveness of interventions in real-world settings as well as generalizability to the larger population (Smith et al., 2007), helping to build the evidence base for an intervention. In order to better understand which telehealth interventions are effective, for whom, and for what outcomes, it is important to examine interventions that use group designs. While some existing reviews (e.g., Ferguson et al., 2019) examine both single-subject and group designs, their assessments of group interventions are limited by virtue of comparing them to SSRDs, which are inherently smaller scale and easier to implement. Further, the group designs are the minority in these reviews — for example, only 28% of the studies included in Ferguson et al. (2019) employed a group design. In order to adequately assess the effectiveness and generalizability of telehealth-based ASD interventions, the current study reviews only those interventions that have been rigorously tested in group designs.

## Current Study

The present review evaluates and synthesizes existing group design research on telehealth interventions for children with ASD. Despite the rapidly growing use of telehealth, especially in the current global health crisis, there are few established guidelines detailing the best practices for implementation. In order to better understand the effectiveness of telehealth in ASD intervention, this review examines studies that were tested using experimental group designs. Improvements in our collective understanding and application of telehealth could have a profound impact on numerous healthcare fields and may play a significant role in achieving equitable distribution of healthcare services.

## Method

### Search Strategy

A comprehensive search for telehealth-based ASD interventions was first conducted in April 2020 using three electronic databases (PsycINFO, PubMed, and Educational Resources Information Center (ERIC)) in psychology, medicine, and education. Search terms included those related to ASD (autism, ASD, autism spectrum disorder, or autis*) and those related to telehealth interventions (telehealth, telemedicine, telepractice, teleconsultation, telepsychiatry, elearning, e-learning, distance learning, online training, remote learning, remote consultation, or videoconferenc*). There were no restrictions placed on the publication years of the search conducted in April 2020, which yielded 532 results. After removing duplicates, 448 articles remained.

A subsequent search using the same search terms was conducted in January 2021, limiting the publication dates to April 2020 through January 2021, in order to identify additional relevant articles published since the previous search. This search yielded 112 results, 30 of which were duplicates, resulting in 82 articles from the second search.

### Screening

The 448 articles from the initial search in April 2020 were independently screened by the first and second authors by title and abstract for inclusion criteria. To be included, articles needed to be peer-reviewed, be published in English, examine an evidence-based intervention for ASD, involve intervention delivery via telehealth, and use a between-groups comparison in the analyses. Determination of what was considered an evidence-based intervention was based on the Phase 2 report from the National Standards Project of the National Autism Center (2015), which lists established interventions for children, adolescents, and young adults with ASD. Based on evidence of intervention effectiveness, professional judgment, values and preferences of patients and families, and capacity for accurate implementation, the National Standards Project identifies 14 established interventions, including: behavioral intervention, cognitive behavioral intervention, natural teaching, and parent training. In this initial screening, most exclusions were made to articles that did not examine an evidence-based intervention for ASD or did not use a between-groups comparison. After this screening, 20 articles underwent full-text review for eligibility, 7 of which did not meet all inclusion criteria. This resulted in an initial 13 articles for review.

**Fig. 1:**
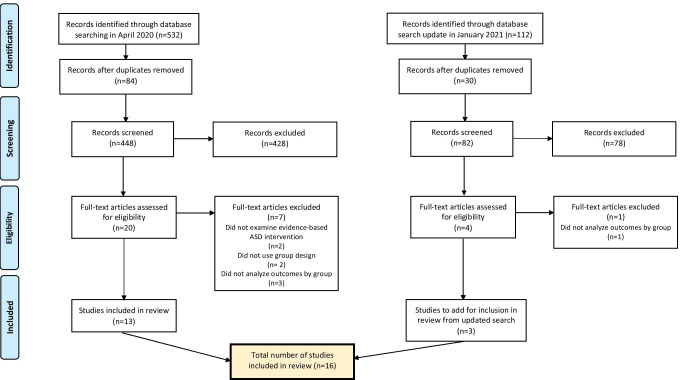
Flow diagram for study selection. From: Moher et al. (2009)

The updated search in January 2021 resulted in an additional 82 articles to be independently screened by the two authors, using the same inclusion criteria described above. This resulted in four additional articles to undergo full-text review for eligibility, one of which did not meet all inclusion criteria, bringing the total number of included studies from both searches to 16. Figure 1 provides an illustration of this selection process.

In both screenings, the first and second authors independently examined the identified studies against exclusion criteria. The results were compared, and inter-rater agreement was determined by dividing the number of agreed eligible studies by the total number of studies and multiplying by 100. Across the two screenings, overall inter-rater agreement between the two authors was 93.6%. Disagreements were resolved by discussion until inter-rater agreement reached 100%.

### Quality Indicators

Methodological quality of the studies was rated using a protocol developed by Reichow et al. (2008), specifically the rubric for evaluating the rigor of group designs. This protocol was chosen over broader metrics (e.g., What Works Clearinghouse, 2018) due to its specificity in evaluating evidence-based practices in the ASD field. Reichow et al. (2008) identify six primary quality indicators for group designs necessary for establishing the validity of a study: descriptions of participant characteristics, independent variables, comparison conditions, dependent variables, links between the research questions and analyses, and statistical data analyses. Primary quality indicators receive ratings of “high,” “acceptable,” or “unacceptable.” This protocol also specifies eight secondary quality indicators of group designs, which are elements considered important to research design but not necessarily critical to study validity. Secondary indicators include randomization, inter-observer agreement, blind raters, generalizability, reporting effect size, and social validity. Secondary indicators are rated on a dichotomous scale (“evidence” or “no evidence”).

Reichow et al. (2008) also specify a method of synthesizing quality indicator ratings into an overall rating of strength of the research report. There are three possible overall ratings: (1) “strong,” in which a study is rated as “high” on all primary quality indicators and shows evidence of at least four or more secondary quality indicators; (2) “adequate,” in which a study receives “high” ratings on four or more primary quality indicators with no “unacceptable” ratings and shows evidence of at least two secondary quality indicators; and (3) “weak,” in which a study receives fewer than four “high” ratings on primary quality indicators and/or shows evidence of fewer than two secondary quality indicators.

Studies were independently assessed by one of the two authors on primary quality indicators, secondary quality indicators, and the overall strength of the report. 43.8% of articles (7 of 16) were assessed by both the first and second authors. These results were compared, and any studies with disagreements in primary quality indicators, secondary quality indicators, and/or overall strength were considered discrepant. Inter-rater agreement was 84.8%, as determined by dividing the number of non-discrepant studies by the number of total studies and multiplying by 100. All disagreements were resolved by discussion until inter-rater agreement reached 100%.

### Data Extraction

Descriptive study characteristics, study design, details regarding the intervention (e.g., intervention implementer, telehealth, and coaching components), child outcomes, implementer outcomes, and summaries of results were extracted by the first author using a customized data extraction form. The second author independently extracted these variables from a randomly selected 37.5% (6 of 16) of the included studies. Inter-rater agreement was 93.0%; disagreements were resolved by discussion until inter-rater agreement reached 100%.

## Results

### Methodological Quality

Overall, the studies included in this review were of mixed quality (see Table 1). Three studies were rated as “strong” (Hepburn et al., 2016; Ruble et al., 2013; Vismara et al., 2018), six were rated as “adequate” (Hao et al., 2021; Ingersoll & Berger, 2015; Ingersoll et al., 2016; Kuravackel et al., 2018; Lindgren et al., 2016; Shire et al., 2020), and seven were rated as “weak” (Blackman et al., 2020; Dai et al., 2018; Fisher et al., 2020; Hay-Hansson & Eldevik, 2013; Marino et al., 2020; Pickard et al., 2016; Vismara et al., 2009).

**Table 1:** Quality ratings of studies of telehealth interventions for children with ASD using group designs

Study	Participant characterstics	IV	Comparison condition	DV	Link between research q and data analysis	Use of stat tests	Random assignment	IOA	Blind raters	Fidelity	Attrition	Generalizability and/or maintenance	Effect size	Social validity	Rating
Blackman et al. (2020)	High	Unacceptable	High	Acceptable	High	High	No evidence	Evidence	No evidence	No evidence	No evidence	No evidence	No evidence	Evidence	Weak
Dai et al. (2018)	Acceptable	High	High	High	High	Acceptable	No evidence	N/A	N/A	No evidence	Evidence	No evidence	No evidence	No evidence	Weak
Fisher et al. (2020)	High	Unacceptable	High	High	High	Acceptable	Evidence	Evidence	Evidence	Evidence	Evidence	No evidence	Evidence	No evidence	Weak
Hao et al. (2021)	Acceptable	High	High	High	High	Acceptable	No evidence	Evidence	Evidence	Evidence	Evidence	No evidence	Evidence	No evidence	Adequate
Hay-Hansson and Eldevik (2013)	High	High	High	High	Unacceptable	Unacceptable	Evidence	Evidence	No evidence	No evidence	No evidence	Evidence	Evidence	No evidence	Weak
Hepburn et al. (2016)	High	Acceptable	High	High	High	High	No evidence	Evidence	N/A	Evidence	Evidence	No evidence	Evidence	No evidence	Strong
Ingersoll and Berger (2015)	High	High	High	Acceptable	High	High	Evidence	No evidence	No evidence	Evidence	Evidence	No evidence	Evidence	No evidence	Adequate
Ingersoll et al. (2016)	High	Acceptable	High	High	High	High	Evidence	Evidence	No evidence	Evidence	No evidence	Evidence	No evidence	No evidence	Adequate
Kuravackel et al. (2018)	High	High	High	High	High	High	Evidence	No evidence	No evidence	Evidence	No evidence	No evidence	Evidence	No evidence	Adequate
Lindgren et al. (2016)	Acceptable	High	High	High	High	High	No evidence	Evidence	No evidence	Evidence	Evidence	No evidence	No evidence	No evidence	Adequate
Marino et al. (2020)	Acceptable	Unacceptable	Acceptable	High	Acceptable	Acceptable	Evidence	No evidence	No evidence	No evidence	Evidence	No evidence	Evidence	No evidence	Weak
Pickard et al. (2016)	Acceptable	High	High	High	High	High	Evidence	Evidence	Evidence	No evidence	Evidence	No evidence	No evidence	No evidence	Weak
Ruble et al. (2013)	High	High	High	High	High	High	Evidence	Evidence	No evidence	Evidence	Evidence	No evidence	Evidence	No evidence	Strong
Shire et al. (2020)	Acceptable	Acceptable	High	High	High	High	No evidence	Evidence	Evidence	Evidence	Evidence	No evidence	Evidence	No evidence	Adequate
Vismara et al. (2018)	High	High	High	High	High	High	Evidence	Evidence	Evidence	Evidence	Evidence	Evidence	No evidence	Evidence	Strong
Vismara et al. (2009)	Acceptable	High	Unacceptable	High	High	Unacceptable	No evidence	Evidence	Evidence	Evidence	Evidence	No evidence	No evidence	No evidence	Weak

Quality indicators retrieved from Reichow et al. (2008). Primary quality indicators include thorough descriptions of participant characteristics, independent variables, comparison conditions, and dependent variables; a strong link between the research question(s) and data analysis; and proper use of statistical analyses. Secondary quality indicators include evidence of random assignment, interobserver agreement, blind raters, fidelity, comparable attrition between groups, generalization and/or maintenance, reports of effect size, and social validity. Strong quality is determined by high quality ratings on all primary quality indicators and evidence of four or more secondary quality indicators. Adequate quality is determined by high quality ratings on four or more primary quality indicators with no unacceptable quality ratings on any primary quality indicators and evidence of at least two secondary quality indicators. Weak quality is determined by fewer than four high quality ratings on primary quality indicators or evidence of less than two secondary quality indicators

**Note: ***IV*, independent variable; *DV*, dependent variable; *IOA*, inter-observer agreement

This group of studies provided thorough descriptions of participant and interventionist characteristics (16 studies); demonstrated a strong link between research questions and data analyses (15); gave replicable definitions of independent variables (13), dependent variables (16), and comparison conditions (15); used proper statistical analyses with adequate power (14); and demonstrated comparable attrition between groups (12). However, many of these studies failed to demonstrate social validity, assess generalization and/or maintenance, and use raters who were blind to the treatment condition. The individual indicator ratings and overall ratings for each study can be found in Table 1.

### Discussion of Study Characteristics

The 16 studies included in this review were summarized and coded for the following elements: study design, intervention, participant characteristics, telehealth setting, telehealth equipment, implementer outcomes, and child outcomes (see Table 2). Each of these elements will now be discussed in detail.

**Table 2:** Summary of studies of telehealth interventions for children with ASD using group designs

Study	Intervention	Study design	N	Child age group	Implementer	Telehealth setting	Telehealth equipment provided	Coaching component	Length of intervention	Child outcome measures	Implementer outcome measures	Summary of findings
Blackman et al. (2020)	ABA principles	RCT: In vivo vs. online self-paced vs. waitlist control. Groups were matched based on pretraining assessment scores	*N* = 18 In vivo (*n* = 7) Online (*n* = 6) Control (*n* = 5)	< 8 yrs	Parents	N/A	N/A	None — parent training only	6 weeks (1×/week, 60–75 min)	Positive parent interactions in play	Positive parent–child play interactions, knowledge assessment, parental stress (PSI-SF), parental competence (PSOC)	Positive parent–child interactions and parent knowledge of ABA increased significantly in both training groups, and the two did not significantly differ from each other. No differences in parental stress or parental competence
Dai et al. (2018)	Parent training program — 6 DVD modules with clips of ABA-based intervention	RCT: treatment group vs. waitlist control group. Groups matched based on clinic locations, child age, gender, and maternal education	*N* = 29 Treatment (*n* = 13) Control (*n* = 16)	18–70 mos	Parents	Home	Research coordinators ensured that participants could access a DVD player	Therapist contacted families for a weekly 15-min phone call	14–16 weeks	None	Self-efficacy (EIPSES), intervention knowledge assessment	Parents rated the program highly and reported that children responded well. Quiz scores for parents in the treatment group increased by 8 points. According to analyses of item-level data, parents in the treatment group became more confident in their parenting abilities post-intervention, while parents in the control group became less confident over time
Fisher et al. (2020)	Parent training program — 9 multimedia modules describing ABA skills; 6 including scripted roleplay	RCT: treatment group vs. waitlist control group. Parents randomly assigned to groups in dyads	*N* = 25 Treatment (*n* = 13) Waitlist control (*n* = 12)	N/A	Parents	Home	Webcam, roleplay materials, Bluetooth headset, and laptop provided when necessary	Researcher provided live coaching while parents roleplayed skills with family member	9 modules (35–60 min) completed at parents’ own pace (between 1.6 and 10.7 mos)	None	Behavioral implementation of skills (BISWA and BISPA), social validity	Treatment group showed marked improvement in correct ABA skills, whereas control group showed small changes. Parents generally rated the virtual training program as socially acceptable
Hao et al. (2021)	SKILLS (based on ImPACT)	Quasi-experimental: parents chose in-person or online training. Groups were matched from a larger population based on child’s age and gender, and maternal education	*N* = 30 On-site (*n* = 15) Videoconference training (*n* = 15)	1–10 yrs	Parents	N/A	N/A	Clinicians guided implementation of strategies while parents interacted with children	8 1-h sessions, including introduction and closing session	Frequency of initiations and responses, number of words, mean length of utterances	Fidelity of intervention implementation	In both groups, parents demonstrated significant increase in fidelity of intervention implementation and children showed significant gains in lexical diversity and morphosyntactic complexity. No significant differences between on-site and videoconference groups
Hay-Hansson and Eldevik (2013)	DTT	RCT: on-site DTT training vs. videoconference training	*N* = 16 On-site (*n* = 8) Videoconference training (*n* = 8)	5–14 yrs	Teachers	Remote sites at schools	Computers and software were provided to remote sites	Experimenter observed teaching sessions and provided feedback or prompting as needed	3 15-min sessions	None	ETE scoring sheet to measure teacher skills	Both groups on avg improved significantly in implementation of DTT at post-test; scores at follow-up decreased somewhat for both groups
Hepburn et al. (2016)	Facing Your Fears (FYF)	Pilot RCT: telehealth intervention vs. waitlist group	*N* = 33 Telehealth intervention (*n* = 17) Waitlist control group (*n* = 16)	7–19 yrs	Parents	Home	N/A	Parents spoke with therapists regularly	12 1.5-h sessions across 3–4 mos	Youth anxiety symptoms (SCARED)	PSOC	Telehealth group showed a significant reduction in youth anxiety symptoms and a significant increase in parenting sense of competence
Ingersoll and Berger (2015)	ImPACT Online	Pilot RCT: Self-directed vs. therapist-assisted telehealth parent training	*N* = 27 Self-directed (*n* = 13) Therapist-assisted (*n* = 14)	27–73 mos	Parents	Home	Families without a personal computer, webcam, or high-speed Internet in the home were provided the necessary technology	Parents in the therapist-assisted group received 2 30-min remote coaching sessions per week (24 total)	Both groups given access to website for 6 mos	None	Intervention fidelity, ImPACT Knowledge Quiz, parent satisfaction survey, parent engagement	Parent engagement and satisfaction was high for both groups, but therapist assistance increased engagement
Ingersoll et al. (2016)	ImPACT Online	Pilot RCT: self-directed vs. therapist-assisted telehealth parent training	*N* = 27 Self-directed (*n* = 13) Therapist-assisted (*n* = 14)	19–73 mos	Parents	N/A (likely home)	N/A, but parents could contact staff with technology-related issues	Parents received 2 30-min live-coaching coaching sessions per week (24 total)	Both groups given access to website for 6 mos	Use of language targets from parent–child interaction, MCDI, VABS-II	Intervention fidelity, PSOC, FIQ	Parents in both groups improved on parent outcomes; parents in the therapist-assisted group had greater gains in fidelity and positive perceptions of child. Children in both groups improved on language measures, but only children in the therapist-group improved in social skills
Kuravackel et al. (2018)	COMPASS for Hope	RCT: in-person vs. telehealth vs. waitlist control	*N* = 33 In-person training (*n* = 13) Telehealth training (*n* = 10) Waitlist control (*n* = 10)	3–12 yrs	Parents	Regional clinic	Equipment available at telemedicine sites	Parents received direct training and support from therapists, either in person or via telehealth	8 weeks — 4 group sessions (2 h), 4 individual sessions (1 h)	Problem behaviors (ECBI)	PSI-4-SF, BPS, CSQ, GSRS	Controlling for pre-treatment behaviors, children in the telehealth condition had fewer parent-rated problem behaviors post-treatment compared to waitlist control; no differences in child outcome between telehealth and in-person condition. No differences in treatment modality on parent outcomes
Lindgren et al. (2016)	FA + FCT	Group comparison: in-home therapy vs. clinic-based telehealth vs. home-based telehealth (not randomized). Child outcomes examined through single-subject analyses	*N* = 94 In-home therapy (*n* = 44) Clinic telehealth (*n* = 20) Home telehealth (*n* = 30)	21–84 mos	Parents	Home, telehealth center/regional clinic	Provided computer equipment to all families	Parents received remote coaching; parent assistants managed equipment at clinic sites	FA: 3–5 sessions (5 min/session) FCT: 25 + weeks or until specific outcome criteria met (5 min/session), ~ 60 min per weekly visit	Video coding of problem behavior, manding, task completion	TARF-R	All 3 models successfully reduced problem behavior. Parent ratings of treatment acceptability were consistently high
Marino et al. (2020)	ABA-based intervention	RCT: Tele-assisted group vs. control group. Randomized block design used to balance groups on gender, age, and developmental quotient	*N* = 42 parents Tele-assisted (*n* = 22) Control (*n* = 20)	3–10 yrs	Parents	Home	N/A	Parents received 1:1 behavioral training and coaching, either in person or via telehealth	12 weeks — 2 h/week of 1:1 training and coaching	Severity of disruptive and noncompliant child behavior, as assessed by parents	PSI-SF	Tele-assisted intervention had a significant positive effect on parents’ stress levels, perception of disruptive and noncompliant behavior of their children, coping with children’s behaviors, and influence on children’s behavior
Pickard et al. (2016)	ImPACT Online	RCT: Self-directed vs. therapist-assisted telehealth parent training	*N* = 28 Self-directed (*n* = 13) Therapist-assisted telehealth (*n* = 15)	19–73 mos	Parents	N/A	N/A	Parents received 2 30-min live-coaching coaching sessions per week (24 total)	12 lessons (length of time N/A)	Perceived child social communication gains (parent survey)	Surveyed intervention acceptability, burden on the family, frequency of program use	Parents in therapist-assisted group showed higher treatment acceptability ratings and perceived greater improvements in child social communication
Ruble et al. (2013)	COMPASS	RCT: COMPASS with face-to-face coaching, COMPASS with web coaching, control	*N* = 44 Placebo control (*n* = 15) Face-to-face coaching (*n* = 14) Web coaching (*n* = 15)	3–9 yrs	Teachers	School	Laptop, webcam, headphones, video camera	4 1.5-h coaching sessions (web or face-to-face) — once every 5 weeks; 30-min technology training for web group prior to coaching	School year	Child progress on IEP goals (PET-GAS)	Teacher adherence to implementation of teaching plants	Students with teachers in the web-based coaching group made greater improvements in goal attainment than students with teachers in the placebo control group; no differences between web and face-to-face groups in student or teacher outcomes
Shire et al., (2020)	JASPER	RCT: face-to-face training vs. remote training	*N* = 27 Face-to-face (*n* = 16) Remote training (*n* = 11) (*N* = 50 children)	2–8 yrs	Interventionists	Home or clinic	N/A	Weekly real-time support	3 mos	JA & BR (ESCS), play acts (SPA)	Implementation fidelity from TCX	No differences in interventionist fidelity (or fidelity improvement) between face-to-face and remote training groups. All children made improvements in IJA and IBR but no group differences. Children with therapists in face-to-face group had greater improvement in total play types
Vismara et al. (2018)	P-ESDM	RCT: telehealth P-ESDM vs. community telehealth TAU	*N* = 24 P-ESDM (*n* = 14) Community telehealth TAU (*n* = 10)	18–28 mos	Parents	Home	N/A	12 weekly 1.5-h video-conferencing sessions — discussion + practice with coaching	12 weeks	Social comm. behaviors (verbal utt., nonverbal JA, imit. functional play)	Treatment fidelity, program website use, satisfaction	P-ESDM group had greater fidelity gains and higher program satisfaction than the community telehealth group. Children in both groups improved in social communication (no group differences)
Vismara et al. (2009)	ESDM	Quasi experimental, effectiveness trial of ESDM and parent coaching for ESDM	*N* = 10 In-person (*n* = 5) Remote (*n* = 5)	12–60 mos	Interventionists (and parents)	Telehealth equipped facilities	N/A	No live coaching of intervention Training: (1) self-instruc., (2) didactic trainings (case discussion, practice), (3) team supervision (discussion, feedback)	10 mos (5 mos direct intervention, 5 mos parent coaching)	Freq of child communic. behaviors (verbal utt., imit. play, imit. verbal utt.), attn. and social init. (CBRS)	Treatment fidelity (therapist and parent), therapist satisfaction	No differences between in-person vs. remote training conditions on ESDM fidelity, parent fidelity, satisfaction with training, child outcomes. Child outcomes were related to therapist and parent fidelity. Limitation: Time and training step were confounded

**Note: Interventions**. *ABA*, applied behavior analysis; *COMPASS*, Collaborative Model for Promoting Competence and Success; *DTT*, Discrete Trial Training; *ESDM*, Early Start Denver Model; *FA*, functional analysis; *FCT*, functional communication training; *ImPACT*, Improving Parents As Communication Teachers; *JASPER*, Joint Attention, Symbolic Play, Engagement, Regulation; *P-ESDM*, Parent training in ESDM

**Child outcomes**. *BR*, behavior regulation; *CBRS*, Child Behavior Rating Scale; *ECBI*, Eyberg Child Behavior Inventory; *ESCS*, Early Social Communication Scales; *JA*, joint attention; *MCDI*, MacArthur-Bates Communicative Development Inventories; *PET-GAS*, Psychometrically Equivalence Tested Goal Attainment Scaling; *SCARED*, Screening for Childhood Anxiety and Related Emotional Disorders; *SPA*, Structured Play Assessment; *VABS-II*, Vineland Adaptive Behavior Scales-2nd edition

**Implementer outcomes**. *BISWA*, Behavioral Implementation of Skills for Work Activities; *BISPA*, Behavioral Implementation of Skills for Play Activities; *BPS*, Being a Parent Scale; *CSQ*, Consultation Satisfaction Questionnaire; *EIPSES*, Early Intervention Parenting Self-Efficacy Scale; *ETE*, Evaluation of Therapeutic Effectiveness; *FIQ*, Family Impact Questionnaire; *GSRS*, Group Session Rating Scale; *PSI-SF*, Parenting Stress Index-Short Form; *PSOC*, Parenting Sense of Competence; *TARF-R*, Treatment Acceptability Rating Form-Revised; *TCX*, Therapist-Child Interaction

### Study Design

Four studies compared a group receiving a face-to-face intervention with a group receiving the same intervention via telehealth (Hao et al., 2021; Hay-Hansson & Eldevik, 2013; Shire et al., 2020; Vismara et al., 2009). Vismara et al. (2009) trained ten therapists to conduct the ESDM intervention and implement a parent coaching model. Five therapists in distant sites were trained via telehealth technology, while the other five participated in a face-to-face training. Similarly, in Shire et al. (2020), 16 interventionists in urban regions received face-to-face training in JASPER, while 11 interventionists in rural regions received remote support. Finally, Hay-Hansson and Eldevik (2013) randomly assigned school staff members to receive either video conferencing training or on-site training in conducting DTT. Finally, Hao and colleagues (Hao et al., 2021) allowed parents to choose between an in-person or online training group, and matched groups from a greater population based on the child’s age and gender and maternal education.

Five studies compared a group receiving intervention via telehealth to a control group — either a waitlist condition (Dai et al., 2018; Fisher et al., 2020; Hepburn et al., 2016) or a group receiving treatment as usual, or TAU (Marino et al., 2020; Vismara et al., 2018). Hepburn et al. (2016) conducted a telehealth version of the Face Your Fears (FYF) intervention with 17 families, comparing the results with secondary data from 37 families who qualified for inclusion but waited at least 3 months before receiving the FYF intervention. Dai et al. (2018) assigned 13 parent–child dyads to a treatment group, who received access to a DVD parent training program, and 16 dyads to a waitlist control group. Fisher and colleagues (Fisher et al. 2020) randomized parents to the treatment group or the waitlist control group in dyads in the order of enrollment. Marino and colleagues (Marino et al., 2020) used a randomized block design to assign participants to a tele-assisted group or a control group while balancing gender, age, and developmental quotient. Lastly, Vismara et al. (2018) randomly assigned 14 parents to a treatment group, where they received an ESDM parent coaching intervention (P-ESDM) via telehealth, and 10 parents to a comparison group. The comparison group received monthly videoconferencing sessions and access to a generic website designed to reflect TAU services in their communities.

Three studies included a face-to-face intervention group, a telehealth intervention group, and a control group (Blackman et al., 2020; Kuravackel et al., 2018; Ruble et al., 2013). Ruble et al. (2013) randomized teacher–child dyads into a placebo control condition receiving online autism training, face-to-face coaching sessions in the COMPASS intervention, or web-based COMPASS coaching sessions. Kuravackel et al. (2018) randomly assigned parents or caregivers to receive the C-HOPE intervention via telehealth, to receive C-HOPE face-to-face, or to be in a waitlist control group. In Blackman et al. (2020), parent–child dyads were assigned to groups based on a pre-training assessment to ensure that each group had similar initial training abilities. Seven parent–child dyads were assigned to receive face-to-face parent training sessions, six dyads were assigned to receive online parent training sessions, and five dyads were assigned to a waitlist control group.

Lindgren et al. (2016) retroactively compared data from groups that received in-home therapy, a clinic-based telehealth intervention, or a home-based telehealth intervention. The in-home therapy group consisted of 52 families with ASD or other developmental disabilities treated between 1996 and 2009. The clinic-based telehealth intervention group included 23 children with ASD treated between 2009 and 2012. The home-based telehealth intervention group included 32 children with ASD treated between 2012 and 2014.

Three studies compared two different telehealth conditions (Ingersoll & Berger, 2015; Ingersoll et al., 2016; Pickard et al., 2016). These articles describe the same trial, in which parents were randomly assigned to receive a self-directed or therapist-assisted version of the ImPACT Online intervention.

### Intervention

The studies in this review employed seven different telehealth-delivered interventions, ranging from more structured interventions to those that were more naturalistic and developmentally oriented: applied behavior analysis (ABA); Face Your Fears (FYF); Collaborative Model for Promoting Competence and Success (COMPASS); Improving Parents as Communication Teachers (ImPACT) Online; Skills and Knowledge of Intervention for Language Learning Success (SKILLS); Joint Attention, Symbolic Play, Engagement, and Regulation (JASPER); and the Early Start Denver Model (ESDM).

#### Applied Behavior Analysis (ABA)

Six studies (Blackman et al., 2020; Dai et al., 2018; Fisher et al., 2020; Hay-Hansson & Eldevik, 2013; Lindgren et al., 2016; Marino et al., 2020) developed parent training interventions focused on basic principles of ABA (Baer et al., 1968), an approach built on behaviorism.

Dai et al. (2018) designed a home-based video parent training program that reviewed cognitive development, challenging behaviors, ABA strategies, and fundamentals of the Picture Exchange Communication System. The curriculum included training modules, behavior reviews, videos demonstrating positive parenting behaviors, and video vignettes. Similarly, Fisher and colleagues (Fisher et al., 2020) developed a parent training program consisting of nine multimedia modules describing ABA skills, six of which included scripted roleplays.

Blackman et al. (2020) designed six parent training modules on introductions to ASD, ABA, behavior management, challenging behavior, increasing communication, and teaching new skills through natural environment training. The face-to-face intervention group watched these modules in person, while the online intervention group watched pre-recorded videos. Marino and colleagues (Marino et al. 2020) similarly administered one-to-one behavioral parent training and coaching on ASD characteristics, behavioral principles, and ABA skills. The tele-assisted group received this training remotely, while the control group received the training in person.

Hay-Hansson and Eldevik (2013) developed an intervention based on discrete trial training (DTT), one of the key teaching methods within ABA (Lovaas & Smith, 2003). The training covered the use of DTT to teach matching, receptive labeling, and expressive labeling. Experimenters first provided information about DTT and modeled two trials, and then provided instructions, modeling, praise, and corrective feedback while the participant practiced with their child. Participants received this training either on-site or via videoconferencing.

Lindgren et al. (2016) trained parents in functional communication training (FCT), another key method used in ABA (Carr & Durand, 1985). Behavior consultants supervised parents as they conducted functional analyses (FA) and FCT. These weekly coaching sessions were conducted via in-home therapy, clinic-based telehealth, or home-based telehealth.

#### Face Your Fears (FYF)

Hepburn et al. (2016) designed a telehealth version of the Face Your Fears intervention (FYF; Reaven et al., 2011), a family-focused, cognitive-behavioral group intervention for anxiety designed for youth with ASD. The first 6 weeks of the intervention reviewed psychoeducational aspects of anxiety; the second 6 weeks promoted the development and implementation of youth-specific anxiety reduction strategies. Parents served as coaches for their children, identifying useful tools and helping their children practice facing targeted fears. The telehealth intervention was individualized to meet the needs of each small group — for example, by providing additional support and modifying homework assignments as needed.

#### Collaborative Model for Promoting Competence and Success (COMPASS)

Two studies used the Collaborative Model for Promoting Competence and Success (COMPASS; Ruble et al., 2012a), either in its original school-based format (Ruble et al., 2013) or in its adapted home-based format, C-HOPE (Kuravackel et al., 2018). Ruble et al. (2013) randomized teachers to a group receiving face-to-face coaching sessions, remote coaching sessions, or to a placebo control. Every 5 weeks, teachers in the treatment groups met with consultants to review videos of teacher-student interactions, score child progress, and discuss teaching plans. Teachers in the face-to-face group met with consultants in person, while teachers in the remote group met with consultants via videoconferencing.

Kuravackel et al. (2018) used the C-HOPE intervention, an outpatient treatment to promote positive parent and child outcomes. Parents participated in both group and individual sessions to review learning differences specific to ASD, evidence-based approaches for managing problem behaviors, and information about parent stress and coping strategies. Parents received intervention at either a university or a regional telehealth center.

#### ImPACT Online

Three articles described a study using the ImPACT Online intervention (Ingersoll & Berger, 2015; Ingersoll et al., 2016; Pickard et al., 2016). ImPACT Online was adapted from Project ImPACT, a naturalistic, developmental-behavioral, parent-mediated intervention for young children with ASD (Ingersoll & Dvortcsak, 2010). Parents in both the self-directed and therapist-assisted groups were given access to the ImPACT Online website, which consisted of 12 lessons. Each lesson contained video clips explaining each technique, a written manual, a self-check quiz, short interactive exercises, and a homework assignment. In addition, parents in the therapist-assisted group received two 30-min remote coaching sessions per week, in which a trained therapist guided them in learning the intervention.

#### Project Skills and Knowledge of Intervention for Language Learning Success (SKILLS)

Hao et al. (2021) designed the Project Skills and Knowledge of Intervention for Language Learning Success (SKILLS) intervention based closely on the ImPACT program (Ingersoll & Dvortcsak, 2010). SKILLS specifically targeted parents’ intervention implementation and children’s communication skills within the context of daily routines and play. This study compared parents receiving the SKILLS program in an online format to those receiving the same intervention in person.

#### Joint Attention, Symbolic Play, Engagement, and Regulation (JASPER)

Shire et al. (2020) delivered training in Joint Attention, Symbolic Play, Engagement, and Regulation (JASPER; Kasari et al., 2006, 2008), an intervention designed to facilitate children’s social engagement, nonverbal and spoken communication, and play skills. Senior trainers completed training in JASPER implementation and coaching. Interventionists were then trained by senior trainers through workshops and practice with weekly feedback. After this training, interventionists each conducted intervention with two children for another 12 weeks while receiving weekly support via either face-to-face or remote meetings.

#### Early Start Denver Model (ESDM)

Two studies implemented the Early Start Denver Model (ESDM; Rogers et al., 2009; Vismara et al., 2009), an intervention for infants and toddlers with ASD (Vismara et al., 2009, 2018). In Vismara et al. (2009), therapists spent 5 months learning the teaching principles, intervention techniques, goal development, data collection methods, and fidelity system of ESDM. They then spent 5 months learning how to educate parents in conducting ESDM. These training sessions were conducted either in person or via telehealth technology. Therapists then practiced the ESDM with families in weekly 1-h treatment sessions for 5 to 6 weeks. Vismara et al. (2018) examined parent training for ESDM (P-ESDM) delivered via telehealth with weekly coaching and access to an ESDM website. P-ESDM was compared to a community TAU telehealth condition, where parents had monthly videoconferencing and access to a website about their child’s community intervention.

### Participant Characteristics

#### Implementer Characteristics

A total of 453 intervention implementers were included across studies, 227 of whom were trained to deliver intervention via telehealth. Ingersoll and Berger (2015), Ingersoll et al. (2016), and Pickard et al. (2016) examined different outcomes from the same trial; therefore, participants from these studies (both adult and child) were counted only once. Parents were the most common implementers of intervention — twelve studies targeted parents as mediators of interventions (Blackman et al., 2020; Dai et al., 2018; Fisher et al., 2020; Hao et al., 2021; Hepburn et al., 2016; Ingersoll & Berger, 2015; Ingersoll et al., 2016; Kuravackel et al., 2018; Lindgren et al., 2016; Marino et al., 2020; Pickard et al., 2016; Vismara et al., 2018). Two studies (Hay-Hansson & Eldevik, 2013; Ruble et al., 2013) targeted teachers in classroom settings. Two studies (Shire et al., 2020; Vismara et al., 2009) targeted therapists as implementers over the course of the telehealth intervention — though it should be noted that Vismara et al. (2009) initially used telehealth to train therapists, who subsequently coached parents in the intervention.

#### Child Characteristics

Children in the included studies ranged in age from 12 months to 19 years (*N* = 451 children). Eleven studies targeted children ranging from early to middle childhood — generally from 2 to 9 years old (Blackman et al., 2020; Dai et al., 2018; Hao et al., 2021; Ingersoll & Berger, 2015; Ingersoll et al., 2016; Lindgren et al., 2016; Marino et al., 2020; Pickard et al., 2016; Ruble et al., 2013; Shire et al., 2020; Vismara et al., 2009). One study (Vismara et al., 2018) only included toddlers 18–28 months in age. Age ranges for three other studies started in childhood and extended into adolescence (5–14 years, Hay-Hansson & Eldevik, 2013; 7–19 years, Hepburn et al., 2016; 3–12 years, Kuravackel et al., 2018). All children had an ASD diagnosis. One study (Fisher et al., 2020) did not include a child group at all, but rather had parents practice the intervention with an unspecified family member.

### Telehealth Setting

The telehealth setting was reported in twelve studies. Five studies state that the intervention took place at home (Dai et al., 2018; Fisher et al., 2020; Hepburn et al., 2016; Ingersoll & Berger, 2015; Vismara et al., 2018), though presumably the intervention described by Ingersoll et al. (2016) and Pickard et al. (2016) did as well. Three other studies took place in either the home or clinic/telehealth center setting, depending on which condition participants were randomized to (Lindgren et al., 2016; Marino et al., 2020; Shire et al., 2020). Telehealth training in Kuravackel et al. (2018) took place at a regional telehealth center. Telehealth-trained therapists in Vismara et al. (2009) were trained at a telehealth equipped facility, but the parent training portion of this study took place in person for both the telehealth and face-to-face groups. Two studies took place in the school setting (Hay-Hansson & Eldevik, 2013; Ruble et al., 2013).

### Telehealth Equipment

Six studies (Dai et al., 2018; Fisher et al., 2020; Hay-Hansson & Eldevik, 2013; Ingersoll & Berger, 2015; Lindgren et al., 2016; Ruble et al., 2013) clearly described the provision of equipment to intervention implementers. Equipment provided generally included a computer, webcam, and/or necessary hardware (e.g., DVD player, video camera, headphones) or software, especially if participants did not have access to these. Ingersoll and Berger (2015) also described providing access to high-speed Internet based on participant need. Presumably these same provisions were made in Ingersoll et al. (2016) and Pickard et al. (2016), though not stated. Pickard et al. (2016) did, however, mention that participants were invited to contact research staff with any technology-related issues. Two additional studies (Kuravackel et al., 2018; Vismara et al., 2009) reported that computer equipment was available at their telehealth sites.

### Implementer Outcomes

All of the studies in this review included measures of implementer outcomes. These outcomes included elements related to learning and implementation of the intervention (e.g., knowledge of the intervention, implementer-child interactions, implementation of the intervention, engagement in the intervention, fidelity), elements related to feasibility and acceptability of the intervention, and elements related to self-perception (e.g., sense of competence, stress).

#### Intervention Knowledge

Implementer knowledge of the relevant intervention was as an outcome measure in three studies (Blackman et al., 2020; Dai et al., 2018; Ingersoll & Berger, 2015). All were assessed pre- and post-intervention using multiple-choice quizzes created from material taught in the respective interventions. Implementers who received training increased in intervention knowledge across all three studies. Control groups in Dai et al. (2018) and Blackman et al. (2020) did not improve in intervention knowledge. There were no differences in intervention knowledge based on treatment modality, either in-person vs. telehealth (Blackman et al., 2020) or self-directed vs. therapist-assisted (Ingersoll & Berger, 2015).

#### Implementer-Child Interactions

Blackman et al. (2020) used implementer-child interactions as an outcome measure, recording the frequency of positive and negative parent–child interactions during a 5-min play session pre- and post-intervention. The proportion of positive interactions significantly increased from pre- to post-intervention for parents receiving face-to-face training and those receiving online training, but there were no significant improvements for parent–child dyads in the waitlist control group.

#### Intervention Implementation

Fisher and colleagues (Fisher et al., 2020) used the Behavioral Implementation of Skills for Work Activities (BISWA; Fisher et al., 2014) and Behavioral Implementation of Skills for Play Activities (BISPA; Fisher et al., 2014) to assess intervention implementation skills. Observers scored whether or not parents correctly implemented an intervention skill at each opportunity for implementation. The BISWA examines instruction delivery, responding to correct responses, responding to problem behavior, and prompting; the BISPA focuses on descriptive praise, delivery of reinforcement, and extinction. Parents in the treatment group showed significant increases on both the BISWA and BISPA, while parents in the waitlist control group did not. Researchers also measured the percentage of skills mastered on the BISWA and BISPA; similarly, parents in the treatment group showed significant increases while parents in the control group did not.

#### Engagement

Implementer engagement was used as an outcome measure in two studies, both of which used electronic tracking to calculate metrics of engagement, such as number of logins to the website and average duration of time spent on the website. Ingersoll and Berger (2015) reported that all parents had a high rate of program engagement, although parents receiving the therapist-assisted intervention demonstrated significantly greater engagement, both in terms of number of logins and time spent on the website. Vismara et al. (2018) reported that parents receiving the P-ESDM telehealth intervention were more engaged with the website and with therapists than parents receiving community-based interventions.

#### Intervention Fidelity

Eight studies used implementer (parent, teacher, or therapist) intervention fidelity as an outcome measure (Hao et al., 2021; Hay-Hansson & Eldevik, 2013; Ingersoll & Berger, 2015; Ingersoll et al., 2016; Ruble et al., 2013; Shire et al., 2020; Vismara et al., 2009, 2018). Overall, implementers in telehealth groups appeared to make gains in intervention fidelity across studies.

Five studies coded implementers’ use of intervention strategies from videotaped sessions (Ingersoll & Berger, 2015; Ingersoll et al., 2016; Shire et al., 2020; Vismara et al., 2009, 2018). Three studies (Hao et al., 2021; Shire et al., 2020; Vismara et al., 2009) reported that implementers (therapists and parents, respectively) in telehealth and in-person groups both made significant gains in fidelity, with no differences in improvement between groups. Vismara et al. (2018) reported significant differences in proportions of parents who met P-ESDM intervention fidelity at study exit, with more parents in the treatment group reaching fidelity than parents in the community TAU group. In Ingersoll and Berger (2015) and Ingersoll et al. (2016), both therapist-assisted and self-directed groups of parents improved in intervention fidelity across time, although the therapist-assisted group made significantly greater gains in fidelity.

Two studies used live observational assessments of fidelity (Hay-Hansson & Eldevik, 2013; Ruble et al., 2013). In Ruble et al. (2013), independent consultants rated the degree to which teachers followed the recommended plan for each coaching session. Teacher fidelity was between 79 and 90%, with no significant differences between the online and face-to-face intervention groups. Hay-Hansson and Eldevik (2013) used the Evaluation of Therapeutic Effectiveness scoring sheet (ETE; Eldevik et al., 2013), which measures competence in DTT implementation. Average ETE scores for both the in-person group and the telehealth group significantly improved over intervention, which was maintained at follow-up.

#### Satisfaction/Acceptability

Nine studies used implementer satisfaction or treatment acceptability rating as an outcome measure (Dai et al., 2018; Fisher et al., 2020; Hepburn et al., 2016; Ingersoll & Berger, 2015; Kuravackel et al., 2018; Lindgren et al., 2016; Pickard et al., 2016; Vismara et al., 2009, 2018). All nine administered a quantitative survey asking implementers to rate factors such as overall satisfaction, intervention acceptability, and website usability; one study also randomly selected ten parents to complete an additional qualitative interview (Pickard et al., 2016). Implementers reported overall high program satisfaction across studies.

Six studies reported no significant differences in satisfaction between face-to-face and online intervention groups (Hepburn et al., 2016; Ingersoll & Berger, 2015; Kuravackel et al., 2018; Lindgren et al., 2016; Pickard et al., 2016; Vismara et al., 2009). In Vismara et al. (2018), however, parents receiving the P-ESDM telehealth intervention reported significantly higher satisfaction and confidence following the intervention than parents receiving a community-based intervention. Dai et al. (2018) only administered the satisfaction survey to parents in the telehealth treatment group, and the group rated the overall program as acceptable and effective. Ingersoll and Berger (2015) reported a marginally significant effect whereby parents in the therapist-assisted intervention group were more satisfied with the program than parents in the self-directed group. Similarly, Pickard et al. (2016) reported that parents in the therapist-assisted group found intervention content to be more accessible and perceived more improvements in children’s social communication skills than did parents in the self-directed group.

Additionally, parents randomly assigned to the additional qualitative assessment administered by Pickard et al. (2016) participated in a 30–45-min semi-structured interview about their overall perception of the intervention and their experience of support during the intervention. While parents in both the therapist-assisted and self-directed groups reported positive perceptions about the acceptability of intervention techniques, parents in the therapist-assisted group spontaneously endorsed the acceptability of the program more than twice as frequently as parents in the self-directed group.

#### Feasibility

Two studies directly assessed program feasibility (Hepburn et al., 2016; Pickard et al., 2016). Hepburn et al. (2016) tracked families’ attendance, major life changes, and technical difficulties through a participant monitoring form. About 6% of sessions were significantly impacted by technical glitches, and 41% of families were disconnected at least once during the ten session intervention. Pickard et al. (2016) asked select parents about the feasibility of the online intervention during semi-structured interviews. Parents appreciated the flexibility of having access to an online program; in particular, parents in the self-directed group were three times more likely to endorse program flexibility than parents in the therapist-assisted group. Parents in the self-directed group were also nearly twice as likely to emphasize time requirements as a barrier to program participation than those in the therapist-assisted group.

#### Sense of Competence

Five studies used implementer sense of competence or self-efficacy as an outcome measure (Blackman et al., 2020; Dai et al., 2018; Hepburn et al., 2016; Ingersoll et al., 2016; Kuravackel et al., 2018). Three of these used the Parenting Sense of Competence Scale (PSOC; Gibaud-Wallston & Wandersmann, 1978; Ohan et al., 2000) to assess sense of competence (Blackman et al., 2020; Hepburn et al., 2016; Ingersoll et al., 2016). Results were mixed. Hepburn et al. (2016) and Ingersoll et al. (2016) both reported significant changes in parent PSOC scores, indicating that parents’ sense of competence increased over the course of intervention. However, Blackman et al. (2020) found no significant changes in PSOC scores between groups or across time.

Dai et al. (2018) used a revised version of the Early Intervention Parenting Self-Efficacy Scale (EIPSES; Guimond et al., 2008) to assess parents’ perceptions of their competence at baseline and post-intervention. Treatment and control groups did not significantly differ in their change in EIPSES score from baseline to post-intervention, but item level analyses suggested that parents in the treatment group became more confident in their parenting abilities over time, while parents in the control group became less confident over time.

Kuravackel et al. (2018) administered the Being a Parent Scale (BPS; Johnston & Mash, 1989) to measure parents’ views of their own competence. Parents who received the face-to-face intervention and parents who received the telehealth intervention expressed gains in competence pre- to post-intervention, with no significant differences between the two groups.

#### Stress

Parent stress was used as an outcome measure in four studies (Blackman et al., 2020; Ingersoll et al., 2016; Kuravackel et al., 2018; Marino et al., 2020). Three of these studies used the Parenting Stress Index – Short Form (PSI-SF; Abidin, 1995) to measure parent-reported stress (Blackman et al., 2020; Kuravackel et al., 2018; Marino et al., 2020). Marino and colleagues (Marino et al., 2020) found that parents in the tele-assisted condition experienced a significant decrease in stress following the intervention and reported feeling better able to face stress than parents in the in-person condition. Kuravackel et al. (2018) found that parents experienced significant decreases in stress over the course of intervention, with no differences between the face-to-face and telehealth trained groups. On the other hand, Blackman et al. (2020) reported no significant changes in parent stress over intervention.

Ingersoll et al. (2016) used the Family Impact Questionnaire (FIQ; Donenberg & Baker, 1993) to measure parental stress and parental perceptions of the child. Parents in both the self-directed and therapist-assisted groups rated themselves as experiencing less stress at post-intervention, with no significant difference between groups. Parents in the therapist-assisted group reported significantly more positive perceptions of their children at post-intervention than those in the self-directed group, although the self-directed group also showed marginally significant increases in positive perceptions of their children from pre- to post-intervention.

### Child Outcomes

Various child-level outcomes were targeted across the included studies. Child outcomes included language and social communication, adaptive skills, reduction in challenging behavior, play skills, and IEP goal progress. Four studies in this review did not include measures of child outcomes. While Lindgren et al. (2016) included three child-level outcomes in their study (problem behavior, manding, and task completion), the study is not included in this section of the review as child outcomes were examined using single-subject analyses rather than group-level analyses.

#### Language and Social Communication

Children’s language and social communication were targeted as outcomes of telehealth interventions in six studies (Hao et al., 2021; Ingersoll et al., 2016; Pickard et al., 2016; Shire et al., 2020; Vismara et al., 2009, 2018). Of these, five used observational measures to examine these outcomes, three of which coded social communication behaviors from parent–child interactions. These included outcomes such as the child’s spontaneous and prompted use of language targets (Ingersoll et al., 2016); morphosyntactic complexity (Hao et al., 2021); spontaneous, functional, and socially directed verbal utterances (Vismara et al., 2009, 2018); nonverbal initiations of joint attention (Vismara et al., 2018); and attention and social initiations (Vismara et al., 2009, using the Child Behavior Rating Scale; Mahoney & Wheeden, 1998). Meanwhile, Shire et al. (2020) coded initiations of joint attention and requesting from a structured direct assessment (Early Social Communication Scales; Mundy et al., 2003). Children were reported to have made gains in observable language and social communication outcomes across studies. Only Ingersoll et al. (2016) found a marginally significant time by treatment group interaction, such that children in the therapist-assisted group made marginally more gains in language target use over those in the self-directed group. There were no other reported group differences based on treatment modality on these outcomes.

Two studies used parent-reported measures to assess changes in child language and communication. Ingersoll et al. (2016) reported parent-reported changes in children’s expressive vocabulary using the MacArthur-Bates Communicative Development Inventory (Fenson et al., 2006). Pickard et al. (2016) used a 49-item parent survey to assess several outcomes, including perceived child social communication gains. Parents reported child language and communication improvement in both studies; only Pickard et al. (2016) reported differences by treatment group over time. Parents in the therapist-assisted telehealth training group reported greater perceived child social communication gains than those in the self-directed training group.

#### Adaptive Skills

Ingersoll et al. (2016) examined parent-reported adaptive skills using the Vineland Adaptive Behavior Scales, second edition (VABS-II; Sparrow et al., 2005). The VABS-II is a standardized parent interview that covers various domains, including communication, daily living skills, socialization, and motor skills. Children in both the therapist-assisted and self-directed groups made gains in the VABS-II communication domain over time. However, only children in the therapist-assisted group made significant gains in the social domain, indicating improvement in parent-reported social skills.

#### Behavior

Three studies (Hepburn et al., 2016; Kuravackel et al., 2018; Marino et al., 2020) used parent reports of children’s behavior as an outcome. Kuravackel et al. (2018) used the Eyberg Child Behavior Inventory (ECBI; Eyberg & Pincus, 1999), which assesses children’s problem behaviors (e.g., oppositional defiant behavior). Kuravackel et al. (2018) reported reductions in problem behavior over time for all groups post-intervention. No differences by treatment group (in-person vs. telehealth vs. waitlist control) or treatment across time were reported. Hepburn et al. (2016) examined parent-reported youth anxiety symptoms across time using the Screening for Childhood Anxiety and Related Emotional Disorders (SCARED; Birmaher et al., 1999), a checklist for anxiety risk. Parents in the telehealth group reported a significant reduction in their children’s anxiety symptoms compared to a waitlist control group. Marino and colleagues (Marino et al., 2020) used the Home Situation Questionnaire (HSQ-ASD; Chowdhury et al., 2016) to assess severity of disruptive and noncompliant child behavior, as reported by parents. Parents in the tele-assisted group reported a significant decrease in their child’s disruptive behavior.

#### Play

Three studies used play skills as child-level outcomes of intervention (Shire et al., 2020; Vismara et al., 2009, 2018). Shire et al. (2020) examined increases in play skills using the Structured Play Assessment (Ungerer & Sigman, 1981), which is designed to measure spontaneous play across various developmental play levels (e.g., simple play to symbolic play). Discrete spontaneous play acts and levels were coded by observers. While children with therapists in both the face-to-face and remote training groups showed improvements specifically in play types (i.e., higher play diversity) and symbolic play types, children with therapists in the face-to-face group showed slightly greater improvement in total play types. There were no differences between treatment groups in improvements in symbolic play.

Vismara et al. (2009) and Vismara et al. (2018) coded children’s play acts from parent–child free play. Imitated functional play (with and without objects) completed within 3 s of a parent’s modeled actions was coded. Vismara et al. (2009) reported that imitation (which also included imitated verbal utterances) did not change over the course of intervention, nor did it differ based on treatment group (telehealth vs. face-to-face training for interventionists, who then trained parents in the intervention). On the other hand, Vismara et al. (2018) reported that children with parents in the telehealth treatment group (P-ESDM) had higher rates of imitation compared to children in the community TAU telehealth group.

#### Progress Toward IEP Goals

Ruble et al. (2013) conducted a school-based intervention, in which student progress on IEP goals was measured. As IEP progress is individualized based on each student’s goals and skills, student progress was measured using Psychometrically Equivalence Tested Goal Attainment Scaling (PET-GAS; Ruble et al., 2012b), which allowed for between-groups comparability. Coders independently rated goals that teachers demonstrated during instructional observations. Results indicate that students with teachers in the web-based coaching group made greater improvements on the PET-GAS than students with teachers in the placebo control group. There were no differences between the web and face-to-face coaching groups.

## Discussion

This literature review provides insight into the rapidly expanding field of telehealth as a means for ASD treatment. As telehealth technology becomes increasingly common, there is a need for large-scale research on its use in the field of ASD (Boisvert et al., 2010; Ferguson et al., 2019; Knutsen et al., 2016; Tomlinson et al., 2018). This review suggests that implementer-mediated ASD interventions executed within a telehealth model can have significant positive outcomes for both implementers and children. Trials that compared telehealth-based interventions to a control condition favored the telehealth condition on both implementer and child outcomes, indicating that telehealth can be an effective way of training others in ASD interventions. This has important implications for intervention delivery, especially for those families that might not have access to high-quality interventions otherwise. Results support previous literature stating that telehealth can be a cost- and time-effective method of disseminating services to the broader ASD population, and that receiving these interventions — even if not delivered in-person, as is traditionally done — improves outcomes over no services or community TAU (Knutsen et al., 2016).

There were generally no significant group differences in outcomes in trials comparing telehealth to a face-to-face intervention. Seeing as interventions varied from more structured approaches (e.g., DTT) to naturalistic developmental behavioral interventions (e.g., ESDM, JASPER, Project ImPACT), this finding suggests that many different types of interventions can effectively be trained and delivered via telehealth. More importantly, this indicates that telehealth-based intervention training can be just as effective as in-person training on a range of outcomes. Notably, fidelity of implementation at the implementer level and social communication outcomes at the child level were comparable across groups.

Two exceptions to this finding were discussed in Blackman et al. (2020) and Shire et al. (2020). Blackman et al. (2020) compared in-person, telehealth, and control conditions on ABA training. Parents in the in-person condition and telehealth condition both had higher positive interactions with their children than controls at the end of intervention; still, there was a significant difference between the in-person and telehealth groups, such that parents in the in-person condition showed greater gains. This may be due to the nature of the telehealth condition in this study (watching pre-recorded videos of training modules without access to a trainer in real-time), which differs from many of the other telehealth conditions included in this review, in which implementers received live coaching and feedback. Shire et al. (2020) compared telehealth vs. face-to-face training of interventionists in JASPER. Children with therapists who were trained in-person made greater improvements in play types over those with therapists trained remotely. JASPER is a modular treatment that utilizes complex intervention strategies, requiring that therapists understand the developmental progression of children’s play and language as well as balance joint engagement and regulation. It may be that there is a specific component of JASPER related to teaching play skills that is better taught and learned in-person, where skills can be modeled in real time.

Several studies compared conditions with varying telehealth delivery models. In those that examined the Project ImPACT Online trial (Ingersoll & Berger, 2015; Ingersoll et al., 2016; Pickard et al., 2016), parents received either self-directed online training or online training with coaching (therapist-assisted training). Parents in the therapist-assisted condition demonstrated gains over those in the self-directed conditions across a range of outcomes. Children in the therapist-assisted intervention group showed greater improvement in adaptive skills and made marginally more gains in social communication outcomes over those in the self-directed intervention group (Ingersoll et al., 2016; Pickard et al., 2016). These findings are consistent with prior research comparing synchronous and asynchronous models of online education delivery. Studies suggest that synchronous models boost motivation and provide more opportunities for learning basic information (Hrastinski, 2008), and are rated by students as more beneficial (Heuberger & Clark, 2019), as compared to asynchronous models. This distinction highlights the importance of coaching, suggesting that telehealth programs should be designed to provide implementers with real-time and ongoing clinician contact when possible. While provision of information is useful, being able to hear feedback, ask questions, and receive support throughout the training process may facilitate greater improvements on both child- and implementer-level outcomes.

### Recommendations for Future Research and Practice

While there has been an increase in group designs of telehealth interventions, there remains a strong need for more large-scale randomized controlled trials. The vast majority of studies examining ASD interventions via telehealth are SSRDs, and the majority of the existing group designs have relatively small sample sizes (Ferguson et al., 2019; Vismara et al., 2009). Therefore, it is difficult to make comparisons between populations or generalize findings based on current literature. As the field of telehealth research grows, robust group designs — particularly those comparing telehealth to another active treatment — are necessary in order to build evidence for this type of intervention delivery. Additionally, assessments of methodological quality revealed a lack of social validity, follow-up assessments, and blind raters. Only 9 out of the evaluated 16 studies (56.3%) reported effect sizes, making it difficult to synthesize quantitative data and compare effectiveness across studies. The addition of these factors in the design of future research would greatly strengthen our understanding of the effectiveness of telehealth interventions.

The majority of articles discussed in this literature review focused on young children, with only three studies including adolescents (Hay-Hansson & Eldevik, 2013; Hepburn et al., 2016; Kuravackel et al., 2018). No studies were specifically designed for children in late elementary, middle, or high school. This underscores an area of need, as well as a gap in our knowledge regarding how effective telehealth interventions may be for these age groups.

Future research conducted on telehealth services should continue to assess a variety of both implementer and child outcomes. Many of the studies included in this review examined treatment acceptability as an outcome for assessing initial feasibility of a treatment; however, other implementer outcomes, such as changes in behavior, attitude, well-being, or fidelity, may become more important as interventions are scaled up and become more widely disseminated. These implementer-level factors may also interact with the effectiveness of the intervention or modality and should be considered a potential mechanism for change in child outcomes.

Additionally, most studies including child outcomes in this review focused on increasing communication skills (e.g., Ingersoll et al., 2016) and decreasing challenging behavior (e.g., Kuravackel et al., 2018), but only Shire et al. (2020) targeted core impairments in ASD such as joint attention (Loveland & Landry, 1986). Researchers and practitioners may consider expanding the scope of telehealth intervention to include interventions targeting such core skills.

Furthermore, much of the research in this field to date has relied on parent report of both parent and child outcomes. As parents are not blind to treatment conditions, this may lead to biased results. While parent reports of satisfaction and perceived support are important, it is crucial that researchers also consider clinician reports of child behavior outcomes and parent implementation outcomes. Corroborating results between parent and clinician reports will likely provide the most accurate results when assessing effectiveness. Future research may also consider parent outcomes as a moderator of the effect of intervention on child outcomes, and vice versa. For example, it is plausible that parents who feel supported and empowered are more successful in implementing interventions with their children. On the other hand, parents may feel more stressed and less motivated to successfully implement intervention if their children are significantly struggling with a particular skill. While these relationships have not been identified in regard to telehealth interventions, they have been seen in other studies of caregiver-mediated ASD interventions (Strauss et al., 2012). Researchers must obtain unbiased data regarding both parent and child outcomes to examine this potential bidirectional relationship.

In addition, telehealth programs should be designed to provide implementers with ongoing clinician contact. While simple training information is important, it is crucial that implementers are also able to hear feedback, ask questions, and receive support throughout their training process. Parents receiving ongoing therapist support demonstrate greater engagement and satisfaction with the intervention (Ingersoll & Berger, 2015) and report greater gains in child outcomes (Ingersoll et al., 2016; Pickard et al., 2016). Having access to a clinician in real-time appears to be an important piece in effective learning and intervention implementation.

As telehealth is a relatively new form of healthcare delivery, little research has examined the aspects of these online interventions that are crucial for long-term success. For example, there are few guidelines as to how intensive and rigorous an online intervention must be to produce the best outcomes. The relatively short-term interventions examined in this review provide promising results, but research on the long-term effectiveness of telehealth interventions is essential going forward.

Lastly, few studies make note of deliberate efforts to make telehealth interventions accessible to a wider range of families, aside from providing access to hardware, software, and the Internet (Dai et al., 2018; Hay-Hansson & Eldevik, 2013; Ingersoll & Berger, 2015; Lindgren et al., 2016; Ruble et al., 2013). Research has identified racial, socioeconomic, and geographic disparities that affect access to diagnostic services and treatment for children with ASD (Liptak et al., 2008; Magaña et al., 2012; Murphy & Ruble, 2012). ASD diagnoses occur later, on average, for African American and Latino children (Mandell et al., 2002), and access to ASD services is limited for racial and ethnic minorities and families with low levels of education (Thomas et al., 2007). It is clear that we must be deliberate about decisions related to the accessibility and effectiveness of these interventions in order to equitably meet families’ needs.

This lack of accessibility must be targeted in research design, recruitment, intervention implementation, and reporting of data. Few studies in this review describe recruitment practices, making it difficult for this review to make claims about recruitment equitability. Going forward, researchers must be intentional about recruiting from underserved communities and reporting these practices. Researchers and interventionists should work directly with community members to develop interventions that are accessible to all families (Jones & Wells, 2007). Policymakers can contribute to the accessibility of services by aiming to support families such as those in underserved communities, those with low incomes, those with low levels of education, and those from minority backgrounds. In actively attempting to reach these families, policy makers, researchers, and interventionists alike may be able to increase family and community health service resources.

Another potential avenue for providing equitable services to children with ASD is to provide these interventions in school settings. As they get older, most children with ASD receive intervention services through their schools (Brookman-Frazee et al., 2009; Sindelar et al., 2010). Two studies (Hay-Hansson & Eldevik, 2013; Ruble et al., 2013) in this review trained teachers to implement interventions with their students, but more are needed in order to maximize access to interventions. Training teachers and other school staff in ASD interventions via telehealth presents an exciting new opportunity to reach students who may not otherwise benefit from these interventions.

### Conclusion

Overall, the expansion of telehealth use in the field of ASD intervention has great potential and has begun to provide services to many underserved individuals. Given the particularly urgent need for increased accessibility to services at the present time, it is crucial that researchers, policymakers, and clinicians continue to assess the quality and accessibility of these telehealth interventions. Through ongoing evaluation and implementation of the recommended strategies, researchers and interventionists will move closer to the goal of equitable access to effective health services for individuals with ASD.

## References

[CR1] Abidin RR (1995). Parenting stress index.

[CR2] Baer DM, Wolf MM, Risley TR (1968). Some current dimensions of applied behavior analysis. Journal of Applied Behavior Analysis.

[CR3] Baharav E, Reiser C (2010). Using telepractice in parent training in early autism. Telemedicine and E-Health.

[CR4] Baio, J., Wiggins, L., Christensen, D. L., Maenner, M. J., Daniels, J., Warren, Z., … Dowling, N. F. (2018). Prevalence of autism spectrum disorder among children aged 8 years – Autism and developmental disabilities monitoring network, 11 sites, United States, 2014. *Morbidity and Mortality Weekly Report,**67*(6), 1–23. 10.15585/mmwr.ss6706a1 10.15585/mmwr.ss6706a1 PMC5919599 29701730

[CR5] Baum A, Kaboli PJ, Schwartz MD (2021). Reduced in-person and increased telehealth visits during the COVID-19 pandemic. Annals of Internal Medicine.

[CR6] Bearss K, Burrell TL, Challa SA, Postorino V, Gillespie SE, Crooks C, Scahill L (2018). Feasibility of parent training via telehealth for children with autism spectrum disorder and disruptive behavior: A demonstration pilot. Journal of Autism and Developmental Disorders.

[CR7] Belfer ML, Saxena S (2006). WHO Child Atlas Project. The Lancet.

[CR8] Birmaher B, Brent DA, Chiappetta L, Bridge J, Monga S, Baugher M (1999). Psychometric properties of the Screen for Child Anxiety Related Emotional Disorders (SCARED): A replication study. Journal of the American Academy of Child and Adolescent Psychiatry.

[CR9] Blackman, A. L., Jimenez-Gomez, C., & Shvarts, S. (2020). Comparison of the efficacy of online versus in-vivo behavior analytic training for parents of children with autism spectrum disorder. *Behavior Analysis: Research and Practice,**20*(1), 13–23. 10.1037/bar0000163

[CR10] Boisvert M, Lang R, Andrianopoulos M, Boscardin ML (2010). Telepractice in the assessment and treatment of individuals with autism spectrum disorders: A systematic review. Developmental Neurorehabilitation.

[CR11] Brookman-Frazee L, Baker-Ericzén MJ, Stahmer AC, Mandell DS, Haine RA, Hough RL (2009). Involvement of youths with autism spectrum disorders or intellectual disabilities in multiple public service systems. Journal of Mental Health Research in Intellectual Disabilities.

[CR12] Carr EG, Durand M (1985). Reducing behavior problems through functional communication training. Journal of Applied Behavior Analysis.

[CR13] Centers for Disease Control and Prevention. (2020). *Implementation of mitigation strategies for communities with local COVID-19 transmission.* Retrieved from https://www.cdc.gov/coronavirus/2019-ncov/downloads/community-mitigation-strategy.pdf.

[CR14] Chowdhury, M., Aman, M. G., Lecavalier, L., Smith, T., Johnson, C., Swiezy, N., … Scahill, L. (2016). Factor structure and psychometric properties of the revised Home Situation Questionnaire for autism spectrum disorder: The Home Situations Questionnaire - Autism Spectrum Disorder. *Autism,**20*, 528–537. 10.1177/1362361315593941 10.1177/1362361315593941 26187059

[CR15] Dai, Y. G., Brennan, L., Como, A., Hughes-Lika, J., Dumont-Mathieu, T., Rathwell, I. C., … Fein, D. A. (2018). A video parent-training program for families of children with autism spectrum disorder in Albania. *Research in Autism Spectrum Disorders,**56*, 36–49. 10.1016/j.rasd.2018.08.008 10.1016/j.rasd.2018.08.008 PMC6605780 31275428

[CR16] Donenberg G, Baker BL (1993). The impact of young children with externalizing behaviors on their families. Journal of Abnormal Child Psychology.

[CR17] Dorsey RE, Topol EJ (2016). State of telehealth. New England Journal of Medicine.

[CR18] Eldevik S, Ondire I, Hughes JC, Grindle C, Randell T, Remington B (2013). Effects of computer simulation training on real life discrete trial teaching. Journal of Autism and Developmental Disorders.

[CR19] Eyberg S, Pincus D (1999). Eyberg child behavior inventory & Sutter-Eyberg student behavior inventory-revised: Professional manual.

[CR20] Fenson L, Marchman VA, Thal D, Dale PS, Reznick JS, Bates E (2006). MacArthur-Bates communicative development inventories: User’s guide and technical manual 2.

[CR21] Ferguson J, Craig EA, Dounavi K (2019). Telehealth as a model for providing behaviour analytic interventions to individuals with autism spectrum disorder: A systematic review. Journal of Autism and Developmental Disorders.

[CR22] Fisher, W. W., Luczynski, K. C., Blowers, A. P., Vosters, M. E., Pisman, M. D., Craig, A. R., … Piazza, C. C. (2020). A randomized clinical trial of a virtual-training program for teaching applied-behavior-analysis skills to parents of children with autism spectrum disorder. *Journal of Applied Behavior Analysis,**53*, 1856–1875. 10.1002/jaba.778 10.1002/jaba.778 32989771

[CR23] Fisher WW, Luczynski KC, Hood SA, Lesser AD, Machado MA, Piazza CC (2014). Preliminary findings of a randomized clinical trial of a virtual-training program for applied behavior analysis technicians. Research in Autism Spectrum Disorders.

[CR24] Gibaud-Wallston J, Wandersmann LP (1978). Development and utility of the parenting sense of competence scale.

[CR25] Guimond AB, Wilcox MJ, Lamorey SG (2008). The early intervention parenting self-efficacy scale (EIPSES): Scale construction and initial psychometric evidence. Journal of Early Intervention.

[CR26] Hao, Y., Franco, J. H., Sundarrajan, M., & Chen, Y. (2021). A pilot study comparing tele-therapy and in-person therapy: Perspectives from parent-mediated intervention for children with autism spectrum disorders. *Journal of Autism and Developmental Disorders,**51*, 129–143. 10.1007/s10803-020-04439-x 10.1007/s10803-020-04439-x 32377905

[CR27] Hay-Hansson, A. W., & Eldevik, S. (2013). Training discrete trials teaching skills using videoconference. *Research in Autism Spectrum Disorders,**7*(11), 1300–1309. 10.1016/j.rasd.2013.07.022

[CR28] Hepburn, S. L., Blakeley-Smith, A., Wolff, B., & Reaven, J. A. (2016). Telehealth delivery of cognitive-behavioral intervention to youth with autism spectrum disorder and anxiety: A pilot study. *Autism,**20*(2), 207–218. 10.1177/1362361315575164 10.1177/1362361315575164 PMC4615367 25896267

[CR29] Heuberger, R., & Clark, W. A. (2019). Synchronous delivery of online graduate education in clinical nutrition: An inquiry into student perceptions and preferences. *Journal of Allied Health, 48*(1), 61–66. Retrieved from: https://pubmed.ncbi.nlm.nih.gov/30826832/. 30826832

[CR30] Hrastinski, S. (2008). Asynchronous and synchronous e-learning. *Educause Quarterly, 4,* 51–55. Retrieved from: https://er.educause.edu/articles/2008/11/asynchronous-and-synchronous-elearning.

[CR31] Ingersoll, B., & Berger, N. I. (2015). Parent engagement with a telehealth-based parent-mediated intervention program for children with autism spectrum disorders: Predictors of program use and parent outcomes. *Journal of Medical Internet Research,**17*(10), e227. 10.2196/jmir.4913 10.2196/jmir.4913 PMC4642401 26443557

[CR32] Ingersoll B, Dvortcsak A (2010). Teaching social communication: A practitioner’s guide to parent training for children with autism.

[CR33] Ingersoll B, Shannon K, Berger N, Pickard K, Holtz B (2017). Self-directed telehealth parent-mediated intervention for children with autism spectrum disorder: Examination of the potential reach and utilization in community settings. Journal of Medical Internet Research.

[CR34] Ingersoll, B., Wainer, A. L., Berger, N. I., Pickard, K. E., & Bonter, N. (2016). Comparison of a self-directed and therapist-assisted telehealth parent-mediated intervention for children with ASD: A pilot RCT. *Journal of Autism and Developmental Disorders,**46*(7), 2275–2284. 10.1007/s10803-016-2755-z 10.1007/s10803-016-2755-z 26922192

[CR35] Johnston C, Mash EJ (1989). A measure of parenting satisfaction and efficacy. Journal of Clinical Child Psychology.

[CR36] Jones L, Wells K (2007). Strategies for academic and clinician engagement in community-participatory partnered research. JAMA: The Journal of the American Medical Association.

[CR37] Kaiser AP, Hancock TB, Nietfeld JP (2000). The effects of parent-implemented enhanced milieu teaching on the social communication of children who have autism. Early Education and Development.

[CR38] Kasari C, Freeman S, Paparella T (2006). Joint attention and symbolic play in young children with autism: A randomized controlled intervention study. Journal of Child Psychology and Psychiatry.

[CR39] Kasari C, Paparella T, Freeman S, Jahromi LB (2008). Language outcome in autism: Randomized comparison of joint attention and play interventions. Journal of Consulting and Clinical Psychology.

[CR40] Kessler, D., Lewis, G., Kaur, S., Wiles, N., King, M., Weich, S., … Peters, T. J. (2009). Therapist-delivered internet psychotherapy for depression in primary care: A randomised controlled trial. *The Lancet,**374*, 628–634. 10.1016/S0140-6736(09)61257-5 10.1016/S0140-6736(09)61257-5 19700005

[CR41] Knutsen J, Wolfe A, Burke BL, Hepburn S, Lindgren S, Coury D (2016). A systematic review of telemedicine in autism spectrum disorders. Review Journal of Autism and Developmental Disorders.

[CR42] Kuravackel, G. M., Ruble, L. A., Reese, R. J., Ables, A. P., Rodgers, A. D., & Toland, M. D. (2018). COMPASS for Hope: Evaluating the effectiveness of a parent training and support program for children with ASD. *Journal of Autism and Developmental Disorders,**48*(2), 404–416. 10.1007/s10803-017-3333-8 10.1007/s10803-017-3333-8 29022130

[CR43] Lindgren, S., Wacker, D., Suess, A., Schieltz, K., Pelzel, K., Kopelman, T., … Waldron, D. (2016). Telehealth and autism: Treating challenging behavior at lower cost. *Pediatrics,**137*(S2), S167–S175. 10.1542/peds.2015-2851O 10.1542/peds.2015-2851O PMC4727312 26908472

[CR44] Liptak GS, Benzoni LB, Mruzek DW, Nolan KW, Thingvoll MA, Wade CM, Fryer GE (2008). Disparities in diagnosis and access to health services for children with autism: Data from the national survey of children’s health. Journal of Developmental and Behavioral Pediatrics.

[CR45] Lovaas O, Smith T, Kazdin AE, Weisz JR (2003). Early and intensive behavioral intervention in autism. Evidence-based psychotherapies for children and adolescents.

[CR46] Loveland KA, Landry SH (1986). Joint attention and language in autism and developmental language delay. Journal of Autism and Developmental Disorders.

[CR47] Machalicek, W., O’Reilly, M., Chan, J. M., Rispoli, M., Lang, R., Davis, T., … Langthorne, P. (2009). Using videoconferencing to support teachers to conduct preference assessments with students with autism and developmental disabilities. *Research in Autism Spectrum Disorders,**3*(1), 32–41. 10.1016/j.rasd.2008.03.004

[CR48] Magaña S, Parish SL, Rose RA, Timberlake M, Swaine JG (2012). Racial and ethnic disparities in quality of health care among children with autism and other developmental disabilities. Intellectual and Developmental Disabilities.

[CR49] Mahoney, G., & Wheeden, C. (1998). Effects of teacher style on the engagement of preschool aged children with special learning needs. *Journal of Developmental and Learning Disorders, 2*(2), 293–315. Retrieved from: http://www.centerforthedevelopingmind.com/sites/default/files/agementofpreschool-agedchildrenwithspeiciallearningneeds.pdf.

[CR50] Mandell DS, Listerud J, Levy SE, Pinto-Martin JA (2002). Race differences in the age at diagnosis among Medicaid-eligible children with autism. Journal of the American Academy of Child & Adolescent Psychiatry.

[CR51] Marino, F., Chilà, P., Failla, C., Crimi, I., Minutoli, R., Puglisi, A., … Pioggia, G. (2020). Tele-assisted behavioral intervention for families with children with autism spectrum disorders: A randomized control trial. *Brain Sciences,**10*, 649. 10.3390/brainsci10090649 10.3390/brainsci10090649 PMC7563357 32961875

[CR52] Meadan H, Daczewitz ME (2015). Internet-based intervention training for parents of young children with disabilities: A promising service-delivery model. Early Child Development and Care.

[CR53] Mervosh, S., Lu, D., & Swales, V. (2020). See which states and cities have told residents to stay at home. *The New York Times.* Retrieved from https://www.nytimes.com/interactive/2020/us/coronavirus-stay-at-home-order.html.

[CR54] Moher D, Liberati A, Tetzlaff J, Altman DG, The PRISMA Group (2009). Preferred reporting items for systematic reviews and meta-analyses: The PRISMA Statement. PLoS Medicine.

[CR55] Mundy P, Delgado C, Block J, Venezia M, Hogan A, Seibert J (2003). A manual for the abridged early social communication scales.

[CR56] Murphy MA, Ruble LA (2012). A comparative study of rurality and urbanicity on access to and satisfaction with services for children with autism spectrum disorders. Rural Special Education Quarterly.

[CR57] National Autism Center (2015). National Standards Project, Phase 2. Retrieved from https://www.nationalautismcenter.org/national-standards-project/.

[CR58] Ohan JL, Leung DW, Johnston C (2000). The parenting sense of competence scale: Evidence of a stable factor structure and validity. Canadian Journal of Behavioural Science.

[CR59] Pickard, K. E., Wainer, A. L., Bailey, K. M., & Ingersoll, B. R. (2016). A mixed-method evaluation of the feasibility and acceptability of a telehealth-based parent-mediated intervention for children with autism spectrum disorder. *Autism,**20*(7), 845–855. 10.1177/1362361315614496 10.1177/1362361315614496 26862084

[CR60] Reaven J, Blakeley-Smith A, Nichols S, Hepburn S (2011). Facing your fears: Group therapy for managing anxiety in children with high-functioning autism spectrum disorders.

[CR61] Reichow B, Volkmar FR, Cicchetti DV (2008). Development of the evaluative method for evaluating and determining evidence-based practices in autism. Journal of Autism and Developmental Disorders.

[CR62] Rogers SJ, Dawson G, Smith CM, Winter JM, Donaldson AL (2009). Early start Denver model intervention for young children with autism manual.

[CR63] Ruble LA, Dalrymple NJ, McGrew JH (2012). Collaborative model for promoting competence and success for students with ASD.

[CR64] Ruble L, McGrew JH, Toland MD (2012). Goal attainment scaling as an outcome measure in randomized controlled trials of psychosocial interventions in autism. Journal of Autism and Developmental Disorders.

[CR65] Ruble, L. A., McGrew, J. H., Toland, M. D., Dalrymple, N. J., & Jung, L. A. (2013). A randomized controlled trial of COMPASS web-based and face-to-face teacher coaching in autism. *Journal of Consulting and Clinical Psychology,**81*(3), 566–572. 10.1037/a0032003 10.1037/a0032003 PMC3744829 23438314

[CR66] Shire, S. Y., Baker Worthman, L., Shih, W., & Kasari, C. (2020). Comparison of face-to-face and remote support for interventionists learning to deliver JASPER intervention with children who have autism. *Journal of Behavioral Education*. 10.1007/s10864-020-09376-4

[CR67] Sindelar PT, Brownell MT, Billingsley B (2010). Special education teacher education research: Current status and future directions. Teacher Education and Special Education.

[CR68] Smith, T., Scahill, L., Dawson, G., Guthrie, D., Lord, C., Odom, S., … Wagner, A. (2007). Designing research studies on psychosocial interventions in autism. *Journal of Autism and Developmental Disorders,**37*(2), 354–366. 10.1007/s10803-006-0173-3 10.1007/s10803-006-0173-3 16897380

[CR69] Snider, M. (2020). What is essential and non-essential during a pandemic? *USA Today.* Retrieved from https://www.usatoday.com/story/money/business/2020/03/21/coronavirus-conditions-lead-state-decisions-essential-businesses/2884758001/.

[CR70] Sparrow SS, Cicchetti DV, Balla DA (2005). Vineland adaptive behavior scales: Second edition (Vineland II), survey interview form/caregiver rating form.

[CR71] Strauss K, Vicari S, Valeri G, D’Elia L, Arima S, Fava L (2012). Parent inclusion in early intensive behavioral intervention: The influence of parental stress, parent treatment fidelity and parent-mediated generalization of behavior targets on child outcomes. Research in Developmental Disabilities.

[CR72] Sutherland R, Trembath D, Roberts J (2018). Telehealth and autism: A systematic search and review of the literature. International Journal of Speech-Language Pathology.

[CR73] Thomas KC, Ellis AR, McLaurin C, Daniels J, Morrissey JP (2007). Access to care for autism-related services. Journal of Autism and Developmental Disorders.

[CR74] Tomlinson SRL, Gore N, McGill P (2018). Training individuals to implement applied behavior analytic procedures via telehealth: A systematic review of the literature. Journal of Behavioral Education.

[CR75] Ungerer JA, Sigman M (1981). Symbolic play and language comprehension in autistic children. Journal of the American Academy of Child Psychiatry.

[CR76] Vismara LA, McCormick C, Young GS, Nadhan A, Monlux K (2013). Preliminary findings of a telehealth approach to parent training in autism. Journal of Autism and Developmental Disorders.

[CR77] Vismara, L. A., McCormick, C. E. B., Wagner, A. L., Monlux, K., Nadhan, A., & Young, G. S. (2018). Telehealth parent training in the Early Start Denver Model: Results from a randomized controlled study. *Focus on Autism and Other Developmental Disabilities,**33*(2), 67–79. 10.1177/1088357616651064

[CR78] Vismara, L. A., Young, G. S., Stahmer, A. C., Griffith, E. M., & Rogers, S. J. (2009). Dissemination of evidence-based practice: Can we train therapists from a distance? *Journal of Autism and Developmental Disorders,**39*(12), 1636–1651. 10.1007/s10803-009-0796-2 10.1007/s10803-009-0796-2 PMC2777219 19582564

[CR79] Wacker, D. P., Lee, J. F., Dalmau, Y. C. P., Kopelman, T. G., Lindgren, S. D., Kuhle, J., … Waldron, D. B. (2013a). Conducting functional analyses of problem behavior via telehealth. *Journal of Applied Behavior Analysis,**46*(1), 31–46. 10.1002/jaba.29 10.1002/jaba.29 PMC5361405 24114083

[CR80] Wacker, D. P., Lee, J. F., Dalmau, Y. C. P., Kopelman, T. G., Lindgren, S. D., Kuhle, J., … Waldron, D. B. (2013b). Conducting functional communication training via telehealth to reduce the problem behavior of young children with autism. *Journal of Developmental Physical Disabilities,**25*(1), 35–48. 10.1007/s10882-012-9314-0 10.1007/s10882-012-9314-0 PMC3608527 23543855

[CR81] Webb, T. L., Joseph, J., Yardley, L., & Michie, S. (2010). Using the Internet to promote health behavior change: A systematic review and meta-analysis of the impact of theoretical basis, use of behavior change techniques, and mode of delivery on efficacy. *Journal of Medical Internet Research*, *12*(1):e4. doi:10.2196/jmir.1376 10.2196/jmir.1376 PMC2836773 20164043

[CR82] What Works Clearinghouse. (2018). Reporting guide for study authors: Group design studies. *Institute of Education Science.* Retrieved from: https://ies.ed.gov/ncee/wwc/Docs/ReferenceResources/wwc_gd_guide_022218.pdf.

